# Novel approaches to analysis of the North Star Ambulatory Assessment (NSAA) in Duchenne muscular dystrophy (DMD): Observations from a phase 2 trial

**DOI:** 10.1371/journal.pone.0272858

**Published:** 2022-08-23

**Authors:** Francesco Muntoni, Michela Guglieri, Jean K. Mah, Kathryn R. Wagner, John F. Brandsema, Russell J. Butterfield, Craig M. McDonald, Anna G. Mayhew, Jeffrey P. Palmer, Shannon Marraffino, Lawrence Charnas, Eugenio Mercuri

**Affiliations:** 1 NIHR Great Ormond Street Hospital Biomedical Research Centre, Great Ormond Street Institute of Child Health, University College London, & Great Ormond Street Hospital Trust, London, United Kingdom; 2 The John Walton Muscular Dystrophy Research Centre, Newcastle University and Newcastle Hospitals NHS Foundation Trust, Newcastle upon Tyne, United Kingdom; 3 Cumming School of Medicine, University of Calgary, Alberta Children’s Hospital, Calgary, Alberta, Canada; 4 Center for Genetic Muscle Disorders, and Departments of Neurology and Neuroscience, Kennedy Krieger Institute, Johns Hopkins School of Medicine, Baltimore, Maryland, United States of America; 5 Division of Neurology, The Children’s Hospital of Philadelphia, Philadelphia, Pennsylvania, United States of America; 6 University of Utah School of Medicine, Salt Lake City, Utah, United States of America; 7 Lawson Health Research Institute, Children’s Hospital, London, Ontario, Canada; 8 Institute of Genetic Medicine, Newcastle University, Newcastle, United Kingdom; 9 Pfizer Inc, Cambridge, Massachusetts, United States of America; 10 Paediatric Neurology, Catholic University, and Centro Clinico Nemo, Fondazione Policlinico Universitario Agostino Gemelli IRCCS, Rome, Italy; PLOS: Public Library of Science, UNITED KINGDOM

## Abstract

**Introduction:**

The North Star Ambulatory Assessment (NSAA) tool is a key instrument for measuring clinical outcomes in patients with Duchenne muscular dystrophy (DMD). To gain a better understanding of the longitudinal utility of the NSAA, we evaluated NSAA data from a phase II trial of 120 patients with DMD treated with domagrozumab or placebo.

**Methods:**

The NSAA exploratory analyses included assessment of individual skills gained/lost, total skills gained/lost, cumulative loss of function, and the impact of transient loss of function due to a temporary disability on NSAA total score (temporary zero score).

**Results:**

There was no significant difference in the total number of NSAA skills gained (mean 1.41 and 1.04, respectively; p = 0.3314) or lost (3.90 vs. 5.0; p = 0.0998) between domagrozumab- vs. placebo-treated patients at week 49. However, domagrozumab-treated patients were less likely to lose the ability to perform a NSAA item (hazard ratio 0.80, 95% confidence interval [CI]: 0.65–0.98, p = 0.029) over 48-weeks vs. placebo-treated patients. When temporary zero scores were changed to “not obtainable” (8 values from 7 patients), domagrozumab-treated patients scored higher on the NSAA total score versus placebo-treated patients (difference at week 49: 2.0, 95% CI: 0.1–3.9, p = 0.0359).

**Conclusions:**

These exploratory analyses reveal additional approaches to interpreting the NSAA data beyond just change in NSAA total score. These observations also highlight the importance of reporting items as “not obtainable” for a patient with a temporary/transient physical disability that impacts their ability to perform the NSAA test.

**ClinicalTrials.gov identifier:**

NCT02310763.

## Introduction

Duchenne muscular dystrophy (DMD) is an X-linked recessive neuromuscular disease caused by loss of function mutations in *DMD*, the gene encoding the dystrophin protein [1]. DMD results in progressive degenerative muscle disease, with early motor delays and clinical signs of muscle weakness recognized in boys aged 3–5 years [2, 3]. Progressive muscle degeneration leads to further muscle weakness and loss of ability to walk by age ~10 years in untreated patients and before ~13 years in glucocorticoid-treated patients [4]. Progressive cardiac complications usually appear in the second decade and, together with respiratory insufficiency, are the leading causes of death in patients with DMD [1, 5, 6].

The North Star Ambulatory Assessment (NSAA) is a 17-item clinician-rated, patient-centered tool that measures ambulatory performance in boys with DMD [7]. Each item can be scored as 0 (unable to perform), 1 (modified method but performed goal independently), or 2 (able to perform independently with no obvious modifications) [8]. The total scores on the NSAA range from 0 to 34, with higher scores indicating better ambulatory function [9]. Assessment of psychometric properties of the NSAA and a Rasch analysis of data from 191 ambulatory boys (3–15 years) have demonstrated that the NSAA is a reliable and valid measure of ambulatory function in patients with DMD [7, 8]. NSAA total score correlates with the 6-minute walk test (6MWT) and other outcome assessments used for patients with DMD [10].

Natural history studies using the NSAA have demonstrated a variability in disease progression and possible prognostic value for predicting progression and loss of ambulation in patients with DMD [11, 12]. To better understand the heterogeneity of disease progression, a study from the UK NorthStar Network used latent class trajectory analysis of NSAA data to describe different groups of boys who share similar trajectories of disease progression [13]. The authors also assessed the probability that a patient would improve, remain stable, or deteriorate on each NSAA item over time [13]. Exploratory analysis of NSAA data from phase III data demonstrated a 31% reduction in risk of losing function across all NSAA items with ataluren versus placebo, an approach that may be more sensitive to demonstrating a treatment effect versus change in total score over time [14].

Following descriptions of alternative analyses for the NSAA [13, 14], and due to the unexpected finding that patients recorded with inability to complete the NSAA (total score = 0) recovered ability in subsequent visits [15], we sought to carry out exploratory analyses to evaluate the effect of treatment on NSAA scores using additional statistical methods. We also reviewed the accuracy of zero NSAA total scores and differentiated these from missing values. More specifically, we evaluated how often a zero on NSAA total score was associated with a temporary disabling condition (such as a sprained ankle or lower limb fracture) and should have been recorded as “not obtainable” compared with irreversible disease progression, for which the total score of zero may be appropriate. Additionally, because many DMD clinical trials used 6MWT as the primary endpoint, we explore the relationship between NSAA total score and 6MWT in domagrozumab-treated patients.

## Methods

### Patients and treatments

Patient and outcome data were extracted from a placebo-controlled, phase II trial of domagrozumab, a myostatin inhibitor (ClinicalTrials.gov: NCT02310763) [15]. The primary efficacy endpoint (change from baseline [CFB] in the time for four-stair climb) was not met, and the totality of evidence did not support significant clinical benefit with domagrozumab, as discussed elsewhere [15]. Change in NSAA total score was a planned secondary endpoint.

Key inclusion criteria included ambulatory boys aged 6 to <16 years, with genetically confirmed DMD, the ability to perform the four-stair climb in 2.5 to ≤12 seconds at screening, treatment with glucocorticosteroids for ≥6 months, and on a stable glucocorticosteroid regimen for ≥3 months prior to screening [15].

The study was a two-period (each 48 weeks), double-blind, placebo-controlled trial where patients were randomized to receive domagrozumab or placebo in one of three sequences groups [15]. Sequence 1, patients received ascending doses of domagrozumab (5, 20, 40 mg/kg) in the first 48 weeks followed by 40 mg/kg in weeks 49–96. Sequence 2, patients received ascending doses of domagrozumab in the first 48 weeks followed by placebo in weeks 49–96. Sequence 3, patients received placebo in the first 48 weeks followed by ascending doses of domagrozumab in weeks 49–96 [15]. For comparison of treatment groups, patients in Sequences 1 and 2 (treated with domagrozumab to week 48) were combined and compared with patients in Sequence 3 (receiving placebo to week 48).

### NSAA

The NSAA was evaluated at screening, baseline, and every 8 weeks thereafter. Mean CFB on NSAA total score was compared for patients treated with domagrozumab versus placebo during follow-up. To evaluate the effectiveness of longer domagrozumab treatment, in the absence of a 96-week placebo arm, mean CFB to week 97 in NSAA total score for patients in Sequence 1 (domagrozumab) was compared with an historical control group from the Cooperative International Neuromuscular Research Group (CINRG) DMD natural history database [4, 16]. From a screened population of 440 in the CINRG historical control group, 40 individuals were matched based on age, glucocorticosteroid usage, baseline four-stair climb time, ambulatory status, and baseline left-ventricular ejection fraction ≥55%. To evaluate if the performance of the placebo group matched the historical control group, mean CFB to week 49 of the NSAA total score for patients in Sequence 3 (placebo) was also compared with this historical control group.

With limited data available at the time of study initiation on the natural history of the NSAA scores using fixed-dosing regimens, an assumption was made that patients who were unable to perform the NSAA at any study visit would not regain the ability on subsequent visits. An assumption was taken that to prevent missing data for patients who attended a scheduled visit, but were unable to perform the NSAA test, a total score of “0” was recorded. Observing a zero NSAA total score at one visit, but then seeing it increase in subsequent visits, created an opportunity to understand how this assumption impacted data interpretation.

In an exploratory sensitivity analysis, we re-evaluated these zero values recorded on NSAA total score to determine whether they were temporarily related to an adverse event (AE) and should have been recorded as “not obtainable.” For this evaluation, we reviewed AEs recorded at the time of the NSAA test to determine whether it was a temporary or permanent physical disability that impacted the patient’s ability to perform the NSAA test. All NSAA total scores of zero were reviewed statistically, and clinically in light of AE data. Any NSAA total score that was recorded as zero but changed to above zero in a subsequent visits was considered a temporary zero and changed to “not obtainable”, if the zero score was associated with an AE and subsequent NSAA scores (ie, once the patient recovered from the AE) stayed above zero. After all applicable temporary zero values were changed to “not obtainable”, the difference in mean CFB to week 49 in NSAA total score between domagrozumab and placebo was recalculated. All applicable patients and AEs are outlined descriptively within the Results.

### Ethics

This trial was conducted in accordance with legal, ethical, and regulatory requirements. The protocol, any amendments, and informed consent documents were approved by the institutional review board or independent ethics committee at each center, namely the Children’s Health Queensland Hospital and Health Service Human Research Ethics Committee, South Brisbane, Australia; The Ethics Committee For Multicenter Trials, Sofia, Bulgaria; the Conjoint Health Research Ethics Board (CHREB, University of Calgary), Calgary, Canada; The Western University Health Science Research Ethics Board, London, Canada; The UBC Children’s and Women’s Research Ethics Board, Vancouver, Canada; The IRB, CHU Sainte-Justine, Montreal, Canada; The Comitato Etico Fondazione Policlinico Universitario A. Gemelli, Rome, Italy; The Comitato Etico IRCCS Ospedale Pediatrico Bambino Gesu, Rome, Italy; The Comitato Etico Regione Liguria Sezione 3 c/o IRCCS Azienda Ospedaliera Universitaria San Martino-IST, Genova, Italy; The National Center of Neurology and Psychiatry Institutional Review Board, Tokyo, Japan; The Hyogo College of Medicine Hospital Institutional Review Board, Hyogo, Japan; The Komisja Bioetyczna przy Warszawskim Uniwersytecie Medycznym, Warszawa, Poland; The NRES Committee Yorkshire and the Humber—Leeds East, Jarrow, United Kingdom; The Washington University in St. Louis, Human Research Protection Office, St. Louis, MO, United States; The Western Institutional Review Board (WIRB), Puyallup, WA, United States; UCLA Medical Institutional Review Board #3 (MIRB3), Los Angeles, CA, United States; Cincinnati Children’s Institutional Review Board, Cincinnati, OH, United States; University of Minnesota Institutional Review Board, Minneapolis, MN, United States; Office of Research, IRB Administration, UC Davis Medical Center, Sacramento, CA, United States; Johns Hopkins Medicine Office of Human Subjects Research Institutional Review Boards, Baltimore, MD, United States; Duke University Health System Institutional Review Board, Durham, NC, United States; University of Utah Institutional Review Board, Salt Lake City, UT, United States; The Children’s Hospital of Philadelphia Institutional Review Board, Philadelphia, PA, United States; The University of Texas Southwestern Medical Center Institutional Review Board, Dallas, TX, United States; Emory University Institutional Review Board, Atlanta, GA, United States; Partners Human Research Committee (PHRC), Someville, MA, United States; PHRC, Boston, MA, United States; Ann and Robert H. Lurie Children’s Hospital of Chicago Institutional Review Board, Chicago, IL, United States; Human Subjects Committee, University of Kansas Medical Center, Kansas City, KS, United States; Chesapeake IRB, Columbia, MD, United States. Parents or legal guardians provided written, informed consent prior to initiation of any trial-related activities.

### Statistical analysis

The preplanned analysis was to assess mean change in NSAA total score from baseline to weeks 17, 33, and 49 for all patients treated with domagrozumab versus placebo [15]. Primary analyses were conducted on the full analysis set (N = 120), which included all randomized patients who received ≥1 dose of study drug. The NSAA total score, including the non-planned sensitivity analysis where transient zero scores were changed to “not obtainable” (as described above), was analyzed using a longitudinal mixed-effects model for repeated measures (MMRM), with baseline four-stair climb time, treatment, and treatment by time interaction as covariates. An additional preplanned analysis included a comparison of the NSAA total score at week 97 with the historical control group (described above).

An exploratory analysis of number of NSAA skills gained on individual items (score change from 0 to 1 or 2) or lost (change from 1 or 2 to 0) from baseline to week 49 was conducted between treatment groups using a non-parametric Wilcoxon rank-sum test. Inconsistencies in the individual item scoring definitions of NSAA items “hop” and “jump” (2 = able; 0, 1 = not able) compared with other items “run 10m” (2 = able, 1 = Duchenne jog, 0 = walk) were not addressed in this analysis.

An additional exploratory analysis to capture cumulative loss of function over time was conducted using a variant of the Andersen-Gill model for repeated events [17]. An event was defined as a patient changing from 2 or 1 at baseline (able to perform a task) to 0 (unable to perform) at any time on any NSAA item. This exploratory analysis included transient zero scores that may have increased to a value above zero at a subsequent visit. A simple linear regression analysis was used to compare CFB to week 49 between NSAA total score and 6MWT, using the Pearson’s product-moment correlation. If patients were physically unable to perform the 6MWT, the distance they walked in meters would be reported as zero. For each of the 8 study visits where the zero NSAA total score was changed to ‘not obtainable’ in the sensitivity analysis, the corresponding 6MWT was not performed, and therefore these visits were not part of the correlation analysis for these patients.

To estimate the minimal detectable change in the NSAA total score, we employed the standard error of measurement distributional approach, as described previously [18]. The effects of patient age, NSAA baseline score, and other potential confounding factors on the analyses were also evaluated. For all exploratory analyses, a *P* value <0.05 was considered statistically significant, without correction for multiple comparisons.

### Data sharing statement

Upon request, and subject to review, Pfizer will provide the data that support the findings of this study. Subject to certain criteria, conditions, and exceptions, Pfizer may also provide access to the related individual de-identified participant data. See https://www.pfizer.com/science/clinical-trials/trial-data-and-results for more information.

## Results

### Prespecified NSAA analyses in the phase II trial

**Fig 1:**
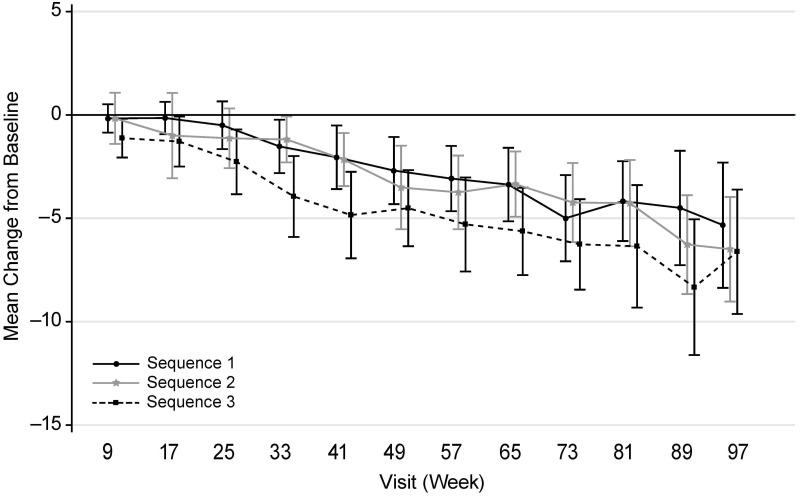
Mean change from baseline in NSAA total score with domagrozumab compared with placebo (full analysis set^a^). CI, confidence interval; NSAA, North Star Ambulatory Assessment. ^a^ all patients who were randomized and received at least one dose of study drug.

In total, 80 subjects were included in the domagrozumab-treatment arm and 40 in the placebo arm (mean±SD age 8.4±1.7 years and 9.3±2.3 years, respectively) [15]. The majority of subjects were White (82.5%, 87.5% respectively) with BMI of 19.4±3.8 and 20.7±5.8 kg/m^2^, respectively [15]. Demographics and baseline characteristics were generally comparable across all sequence groups [15]. Test–retest reliability analysis showed strong correlation between baseline and screening data (*r* = 0.95, p<0.0001). Based on the MMRM analysis, there was no significant difference in mean CFB in NSAA total score with domagrozumab versus placebo at week 49 (1.6; 95% confidence interval [CI]: –0.5, 3.8; p = 0.13; Fig 1) [15].

**Fig 2:**
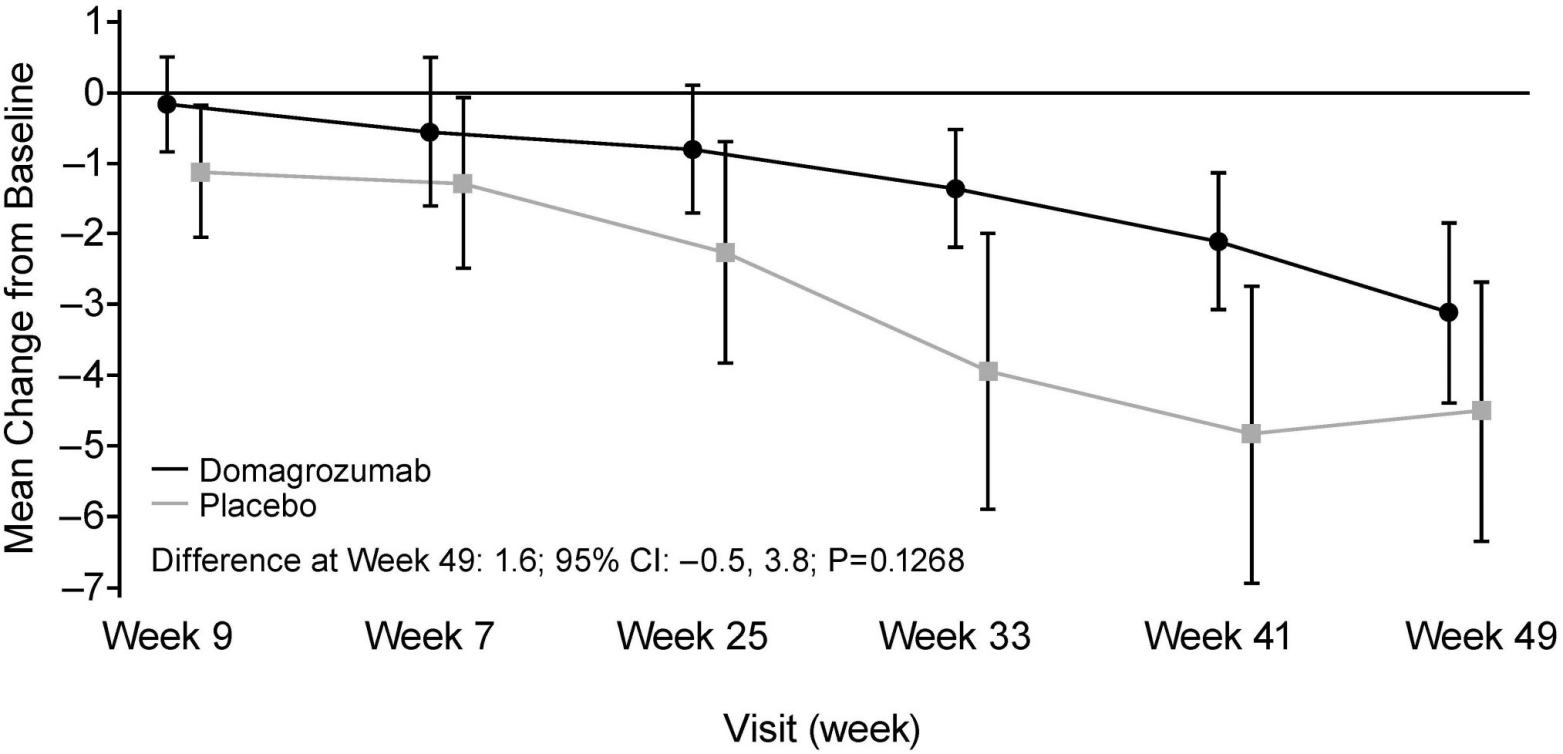
Mean change from baseline in NSAA total score by treatment sequence (full analysis set^a^). *Sequence 1*: Domagrozumab dose escalation (5, 20, 40 mg/kg) in first 48 weeks followed by domagrozumab 40 mg/kg in weeks 49–96. *Sequence 2*: Domagrozumab dose escalation in first 48 weeks followed by placebo in weeks 49–96. *Sequence 3*: placebo in first 48 weeks followed by domagrozumab dose escalation in weeks 49–96. ^a^ all patients who were randomized and received at least one dose of study drug. NSAA, North Star Ambulatory Assessment.

Overall, mean CFB in NSAA total score through to week 97 was comparable for all three treatment sequences (Fig 2). A significant difference was detected in mean CFB to week 97 on the NSAA total score between Sequence 1 (domagrozumab treatment for 96 weeks) compared with the historical control group (difference –3.9; 95% CI: –7.0, –0.8; p = 0.0146) and in mean CFB to week 49 in NSAA total score between Sequence 3 (placebo first 48 weeks) and the historical control group (difference of –2.9; 95% CI: –5.7, 0.0; p = 0.0483).

### NSAA skills gained or lost, and cumulative loss of function

**Fig 3:**
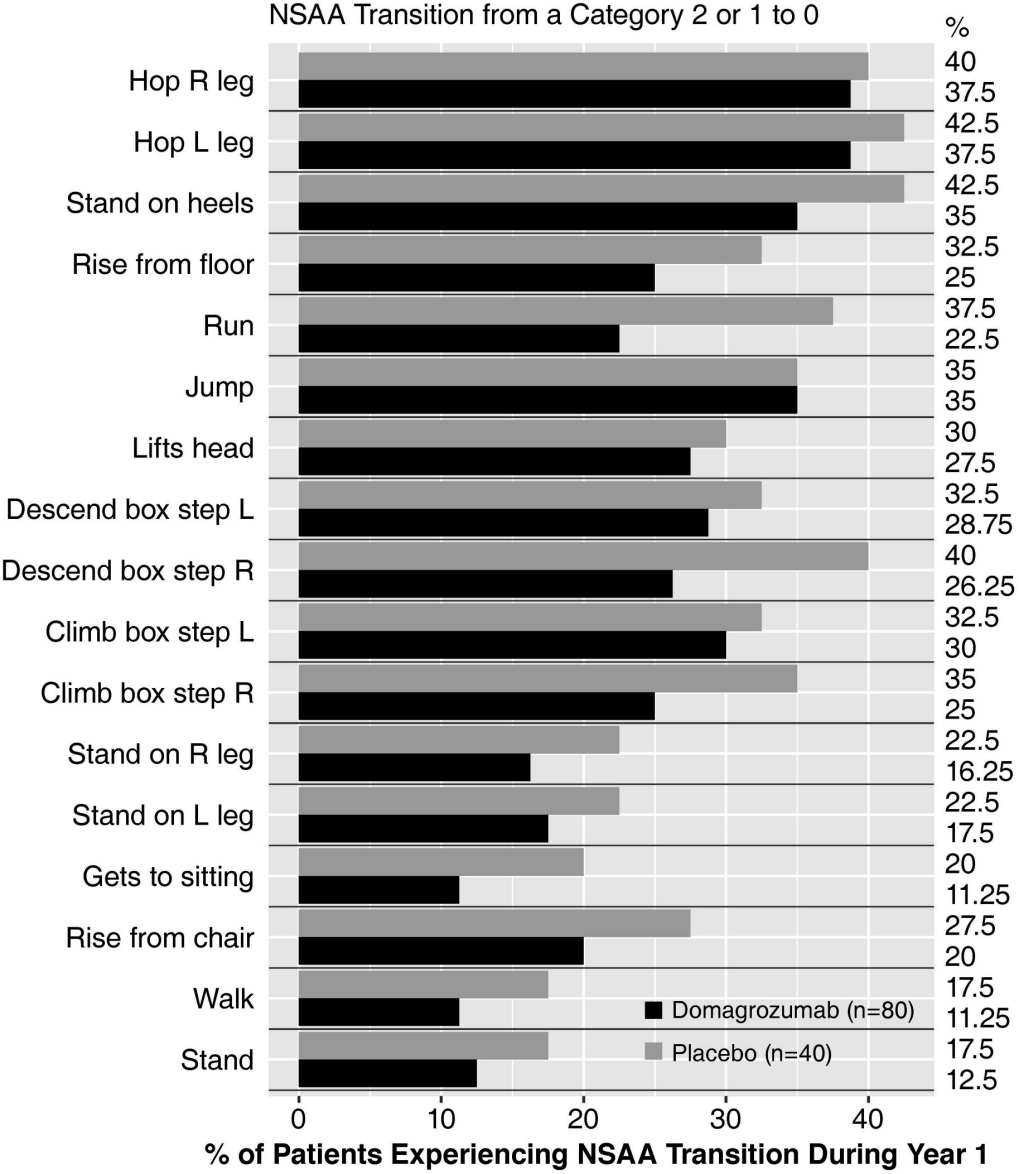
Proportion of patients who lost function in individual NSAA test items (full analysis set^a^). L, left; NSAA, North Star Ambulatory Assessment; R, right. ^a^ all patients who were randomized and received at least one dose of study drug.

The proportion of patients with individual skills lost (item score changed from 2 or 1, to 0) by week 49 for each of the NSAA components was generally lower in domagrozumab versus placebo-treated patients (Fig 3). More specifically, patients treated with domagrozumab lost fewer individual skills over 48 weeks compared with placebo-treated patients, including “gets to sitting”, “run”, “walk”, and “descend box step R” (Fig 3).

**Fig 4:**
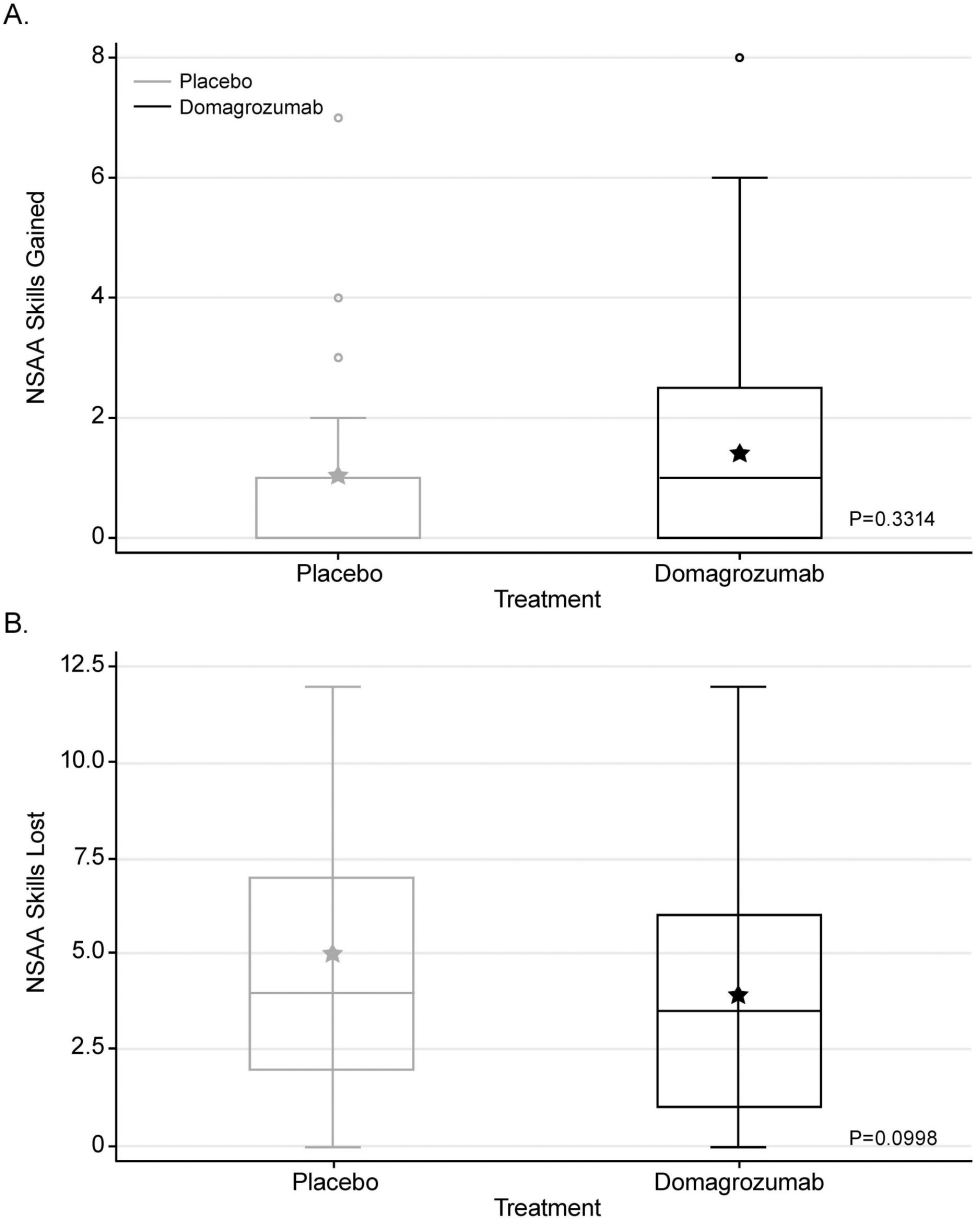
Cumulative number of skills (A) gained or (B) lost, through week 49, in domagrozumab- vs. placebo-treated patients. Data are median [1st and 3rd quartiles]. The stars represent the mean and the circles are the outliers. NSAA, North Star Ambulatory Assessment.

There was no statistical difference between the mean number of skills gained (score change from 0 to 1 or 2, or from 1 to 2) from baseline to week 49 between domagrozumab- and placebo-treated patients (1.41 vs. 1.04, p = 0.3314; Fig 4A). Similarly, there was no statistical difference between the mean number of NSAA skills lost (score change from 2 to 1 or 0, or from 1 to 0) from baseline to week 49 for domagrozumab versus placebo-treated patients (3.90 vs. 5.00, p = 0.0998; Fig 4B).

**Fig 5:**
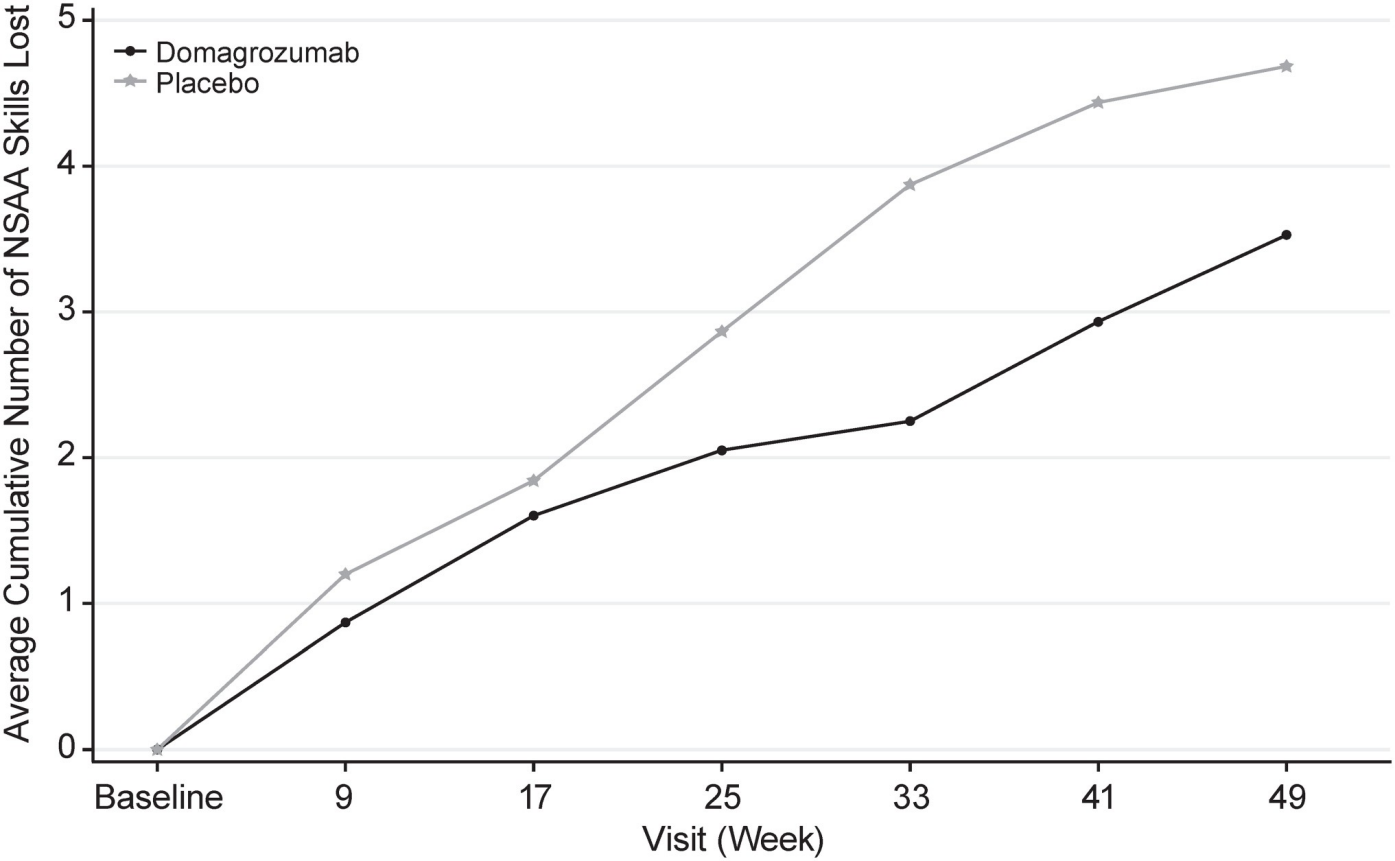
Cumulative loss of individual NSAA skills over 48 weeks (full analysis set). Event was defined as a patient dropping from 2 or 1 (able to perform a task) to 0 (unable to perform). This exploratory analysis included transient zero scores that may have increased to a value above zero at a subsequent visit. NSAA, North Star Ambulatory Assessment.

In an analysis of cumulative loss of function to week 49, domagrozumab-treated patients were 20% less likely to lose the ability to independently perform an NSAA task compared with those receiving placebo (hazard ratio [HR] 0.80; 95% CI: 0.65, 0.98; p = 0.029; Fig 5).

### Investigation of temporary NSAA total score of zero

On review of the NSAA total scores of zero, there were 25 patients with at least one total score of zero recorded during the first 48 weeks. In nine of these 25 patients, the zero scores were evaluated as these patients reverted back to above zero in at least one subsequent clinical visit (Table 1). On examination of these temporary zero scores, eight values (from 7 patients) were changed to “not obtainable,” due to the value being associated with an AE linked to the inability to perform the assessment (Table 1). Individual patient narratives are shown in S1 Fig. Despite at least one NSAA total score being greater than zero following these temporary zero scores in the remaining 2 of 9 patients, the first zero scores were retained because AEs of loss of ambulation were reported simultaneously with these first zero total scores (Table 1), and the NSAA total score was again zero by end of study (S1 Fig, n = 2 patients indicated with red boxes).

**Table 1:** Identified patients with temporary zero value for NSAA total score through week 49.

Treatment (sequence^a^)	Week at 0 score (AE reported)	Week score >0 (score)	Action for sensitivity analysis
Domagrozumab (1)	41 (hip fracture)	49 (7)	Week 41 (0) → not obtainable
Domagrozumab (2)	9 (lower limb fracture)	17 (10)	Week 9 (0) → not obtainable
Domagrozumab (2)	17 (fall and hip fracture)	25 (30)	Week 17 (0) → not obtainable
*Domagrozumab (1)*	*25 and 33 (loss of ambulation)* ^b^	*41 (3)*	*Weeks 25 and 33 → leave as 0* ^b^
Placebo (3)	25 (fall and ligament sprain)	33 (6)	Week 25 (0) → not obtainable
Placebo (3)	41 (femur fracture)	49 (11)	Week 41 (0) → not obtainable
*Placebo (3)*	*49 (loss of ambulation)* ^c^	*57 (6)*	*Week 49 → leave as 0* ^c^
Placebo (3)	33 and 41 (femur fracture)	49 (16)	Weeks 33 and 41 (0) → not obtainable
Domagrozumab (2)	49 (hip fracture)	57 (12)	Week 49 (0) → not obtainable

*Italic* text indicates two patients who had total score of zero retained in sensitivity analyses (as indicated on S1 Fig with red boxes).

^a^Sequence 1: within patient ascending doses (5, 20, and 40 mg/kg) of domagrozumab in the first 48 weeks followed by 40 mg/kg in weeks 49–96. Sequence 2: within patient ascending doses of domagrozumab in the first 48 weeks followed by placebo in weeks 49–96. Sequence 3: placebo in the first 48 weeks followed by within patient ascending doses of domagrozumab in weeks 49–96.

^b^AE of ‘Gain Inability’ was unresolved and recorded as loss of ambulation. Patient had total score of zero retained in sensitivity analyses.

^c^AE of “Loss of ambulation” was unresolved. Patient had total score of zero retained in sensitivity analyses.

AE, adverse event; NSAA, North Star Ambulatory Assessment.

A *post hoc* analysis of mean CFB in NSAA total score, after changing these 8 study visits (from 7 patients) to ‘not obtainable’, reported a statistically significant difference favoring domagrozumab at week 49 (p = 0.0359) (Table 2). Therefore, a significant impact on the overall interpretation of this endpoint was seen.

**Table 2:** Mean change from baseline (CFB) in the NSAA total score (from Week 9 to Week 49). Full analysis and sensitivity analysis, where temporary zero values were changed to “not obtainable”^a^.

Week	Full analysis set				Sensitivity analysis			
	Domagrozumab, mean CFB (SE)	Placebo, mean CFB (SE)	Domagrozumab vs. placebo difference, mean (95% CI)	*P* value	Domagrozumab, mean CFB (SE)	Placebo, mean CFB (SE)	Domagrozumab vs. placebo difference, mean (95% CI)	*P* value
9	–0.8 (0.5)	–1.7 (0.6)	1.0 (–0.2, 2.1)	0.0892	–0.6 (0.4)	–1.8 (0.5)	1.1 (0.2, 2.1)	0.0170
17	–1.1 (0.6)	–1.9 (0.8)	0.8 (–0.9, 2.5)	0.3522	–0.7 (0.5)	–1.9 (0.6)	1.2 (0.0, 2.4)	0.0546
25	–1.4 (0.6)	–2.7 (0.8)	1.3 (–0.3, 2.9)	0.1075	–1.4 (0.5)	–2.5 (0.7)	1.1 (–0.5, 2.7)	0.1651
33	–2.0 (0.6)	–4.5 (0.8)	2.5 (0.7, 4.2)	0.0061	–2.1 (0.5)	–4.0 (0.7)	1.9 (0.4, 3.4)	0.0149
41	–2.7 (0.7)	–5.5 (0.9)	2.9 (0.9, 4.9)	0.0048	–2.6 (0.6)	–4.8 (0.8)	2.2 (0.5, 3.9)	0.0137
49	–3.6 (0.7)	–5.2 (0.9)	1.6 (–0.5, 3.8)	0.1268	–3.3 (0.6)	–5.3 (0.8)	2.0 (0.1, 3.9)	0.0359

^a^ see Table 1 for detail and S1 Fig for patient narratives.

CI, confidence interval; NSAA, North Star Ambulatory Assessment; SE, standard error.

### Relationship between change in NSAA total score and 6MWT, and the minimal detectable change in NSAA total score

A statistically significant correlation was seen in a regression analysis between NSAA total score and 6MWT from this patient population (*r* = 0.50, p<0.0001; S2 Fig).

Using the standard error of measurement statistical distribution method [18], which accounts for reproducibility between screening and baseline visits, the minimum detectable change in NSAA total score was estimated to be 1.52 points.

## Discussion

To gain a better understanding of longitudinal changes in motor function in DMD, we evaluated the NSAA from a phase II trial in 120 boys with DMD treated with domagrozumab or placebo. Similar to the findings of the primary endpoint of four-stair climb time [15], the NSAA total score in the full analysis dataset favored domagrozumab, but results were not statistically significant. However, the purpose of the current analyses was to explore the utility of the NSAA as a tool to detect change in motor function, and ultimately to better understand the responsiveness of the NSAA within a structured clinical trial setting.

To understand the long-term function of the NSAA, we analyzed the mean CFB to week 97 looking at data from Sequence 1 (domagrozumab) and a historical control group. Although a statistically significant difference was seen, this result should be considered with caution because of the significant difference in mean CFB to week 49 between the Sequence 3 placebo group and the historical control group. These observations suggest utilizing the baseline four-stair climb time as the only functional assessment when population matching for the NSAA comparison was inadequate, and further baseline functional scores should be used [19]. Indeed, since the start of the current study on which our data are derived, the discrepancy between trial populations and natural history has been described [20]. Our observations therefore reinforce the need for careful matching of patient criteria when using natural history studies, as identification of more carefully matched untreated patients from a natural history cohort could alleviate the burden/need for a large number of placebo-treated patients. Although it is not possible to match all characteristics, a better understanding of prognostic factors for functional outcomes in DMD will continue to help guide matched or adjusted comparisons to external control groups [19].

Another aspect of our study was to explore the granularity of the NSAA based on individual skills lost or gained. There was no significant difference in the overall number of skills gained or lost over 49 weeks of follow-up for patients treated with domagrozumab versus placebo. Although the treated and untreated population were well-balanced on NSAA total scores at baseline, these observations are potentially confounded by the difference in ages between treatment arms (baseline mean 8.4 vs. 9.3 years, domagrozumab vs. placebo, respectively). The analysis was not adjusted by baseline age, and this can be considered a limitation as since the initiation of this study the impact of age on skills lost and gained has been discussed elsewhere [13]. More specifically, skills gained may be driven by the proportion of younger patients (ie, 6 and 7 years) within each cohort, consistent with natural history data in which the largest improvement was seen in patients younger than seven [13]. Our exploratory analysis of cumulative loss of function over 48 weeks did suggest that patients were 20% less likely to lose the ability to perform an NSAA task when receiving treatment with domagrozumab versus placebo, but given the imbalance of age (noted above) the change may have been driven by younger age rather than treatment effect. However, both treatment and placebo groups were well-balanced on measures of baseline disease severity shown to have equal or greater prognostic significance to age, such as time to stand from supine, time to climb 4-stairs, time to complete 10-m run/walk, and baseline NSAA score [15]. A similar cumulative failures approach has recently been described for the NSAA, with a greater ability to demonstrate a modest treatment effect with ataluren and different steroid regimes in DMD [21].

These data and other reports also suggest differing sensitivities of the proposed shift analysis based on baseline functional status and the specific items, whereby the probability of shift changes over 1 year is different for the different NSAA skills [13, 14, 22]. Other factors, such as where a patient starts at baseline, will also influence change over time, as change is not linear in these patients. Further studies will be needed to confirm these observations. Taken together, these exploratory analyses looking at different ways to assess NSAA skills suggest a more granular approach to functional status, based on individual NSAA skills, may be more sensitive to capturing treatment effects in patients with DMD. Further studies are therefore needed in this area.

The NSAA is a test that can be easily performed in ~10 minutes and used in a multicentric setting, providing that adequate training is given [9]. The impact of any instructions given around patients with a transient inability to perform the NSAA, due to an AE, has not been previously investigated. Our analysis in which temporary zero values related to an AE were changed to “not obtainable” showed statistically significant differences on NSAA total score, whereas the overall analysis was not significant (CFB domagrozumab vs. placebo at week 49). More specifically, for seven patients, eight temporary zero scores recorded during the first 48 weeks were associated with the patients having a transient physical disability, commonly a fracture. This highlights the need for clearer guidance on conducting the NSAA test, particularly in a structured clinical trial environment, when testing a patient who is temporarily unable to perform the required assessments at a given timepoint. Outside the scope of the current study was looking at the same approach within the historical control group, which would require access to the same patient-level visit-by-visit AE data from the matched patients. Ongoing, or future clinical trials where the NSAA is being used should consider the implications of our findings and take into account the limitations of the NSAA highlighted in our study.

Methods of handling missing data are put in place during study design and in the training materials, and individuals recording scores during patient assessments (physicians, physical therapists, etc.) may have attempted to avoid missing data during filling in patient assessments. A conservative approach to scoring ‘0’ for missing or temporarily unattainable data is used in many trial designs intentionally. Indeed, this approach would be predetermined in the protocol, but other approaches to missing data could be proposed, particularly as our sensitivity analyses demonstrate that use of ‘0’ scores influenced the significance of the conclusions. These observations will hopefully inform future trial design in this area, such as use of multiple imputation. For example, instructions for conducting the NSAA should emphasize that if a patient has a fracture and/or temporarily cannot perform, the total score should be recorded as “not obtainable” with clear explanation as to why, rather than total score of zero. The NSAA total score becomes “not obtainable” if any of the 17 items are “not obtainable” on assessment, but the individual items should each be recorded, where possible. Explanations will be key to understanding this “not obtainable” qualification, particularly as it may not be clear at the time of assessment if the patient will recover sufficiently to complete NSAA items in the future. The plan therefore needs to account for a permanent loss of function versus a temporary loss. Clearer guidance on when to stop collecting NSAA data, and perhaps a different definition of ‘non-ambulatory’ within clinical trial documentation, might also help guide individuals *not* to use a zero NSAA total score on any clinical visits if the patient has potential to score on any NSAA tests. This guidance will take away uncertainty, and thus help improve accuracy.

Finally, we also confirmed that the NSAA total score had “good” correlation with 6MWT for patients treated with domagrozumab (correlation of 0.50) and was consistent with previous observations, from 112 boys reporting a coefficient of 0.589 [10]. Based on the current analysis, we also estimate that 1.52 points was the minimal detectable change in NSAA total score. This difference is based on distributional assessment and not related to clinical response, and is broadly similar to findings of a minimum detectable change for NSAA of 2.0 points using the 1/3 SD method [23]. The content validity and patient input used in test construction of the NSAA would imply a loss or gain of a single function to be clinically meaningful. Anchor-based analyses using patient-reported outcome measures should help further determine the changes on the NSAA that are clinically meaningful. Additional studies with a longer follow-up period in various stages of disease, including more transitions of functional events known to be meaningful to patients with DMD and their caregivers, will help define the magnitude of the minimal clinically important difference in the NSAA total score.

## Conclusions

These exploratory analyses of NSAA items, such as of individual skill loss, total skills gained/lost, and time to loss of function, revealed additional approaches to understand the utility of NSAA in patients with DMD that were not apparent when evaluating only CFB in the NSAA total score. If a NSAA item can be performed, it should be recorded. If any activity cannot be performed because of a temporary injury or incapacity, then “not obtainable” should be scored, and the NSAA Total Score becomes “not obtainable” if any of the 17 items are “not obtainable”, as at that time it is not known if the NSAA total score of zero will be temporary. The NSAA assesses a multitude of motor tasks and functions, and each task or function is clinically meaningful. However, further work is required to understand the minimally important clinical differences in these exploratory analyses in order to consider their clinical usefulness in drug development for the treatment of DMD.

## Supporting information

S1 Fig Descriptive assessments in NSAA total score for nine patients with transient zero scores assessed for the sensitivity analysis (see Table 2), shown by treatment sequence. (TIF)

S2 Fig Relationship between change in the NSAA total score and change in 6MWTa, from baseline to week 49. (TIF)
