# Supplementary figures and images for: Novel approaches to analysis of the North Star Ambulatory Assessment (NSAA) in Duchenne muscular dystrophy (DMD): Observations from a phase 2 trial
Source: PLoS One. 2022 Aug 23;17(8):e0272858. doi: 10.1371/journal.pone.0272858 (PMC9397979; doi:10.1371/journal.pone.0272858)

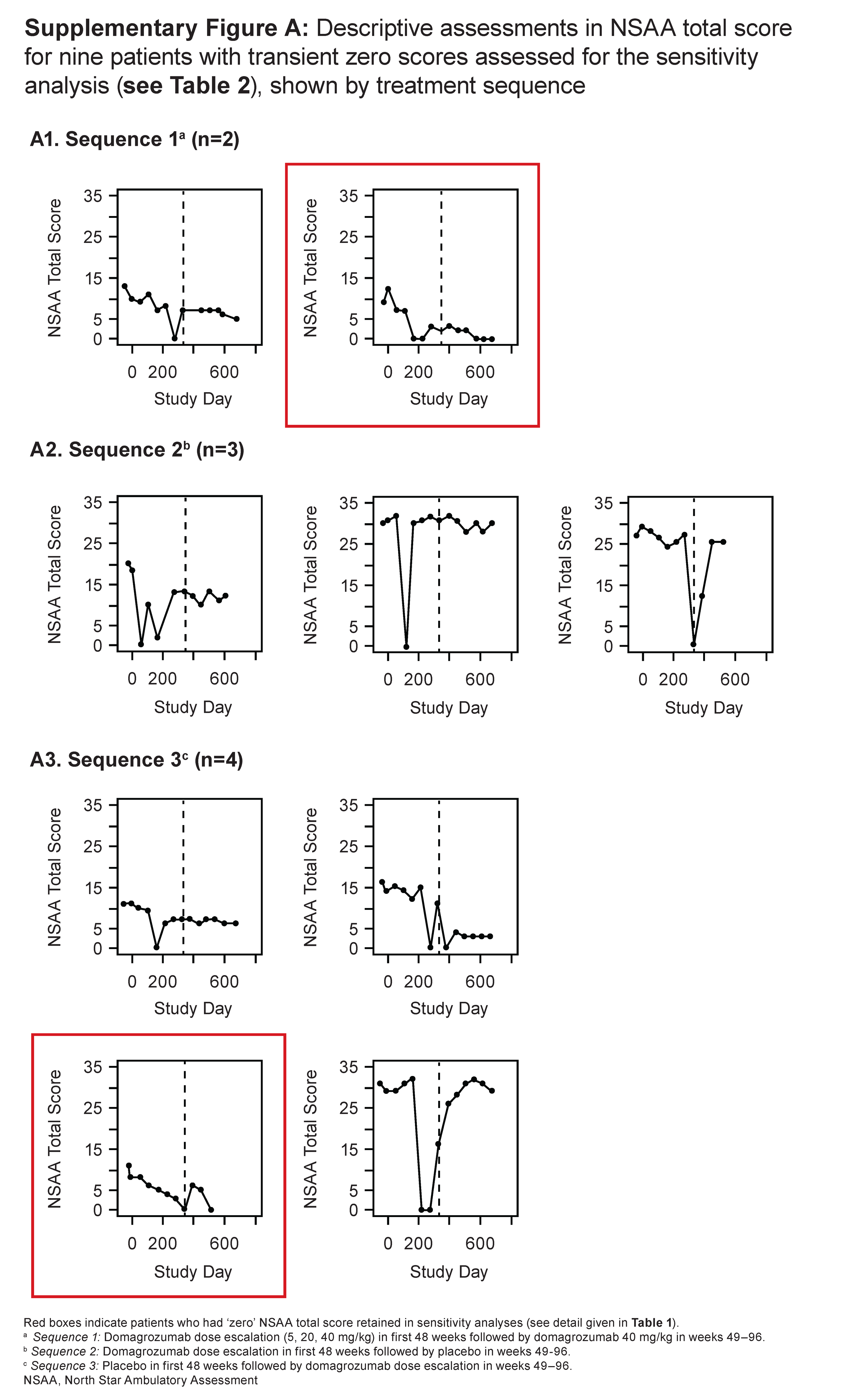

Supplement: S1 Fig — (TIF) [file pone.0272858.s001.tif]

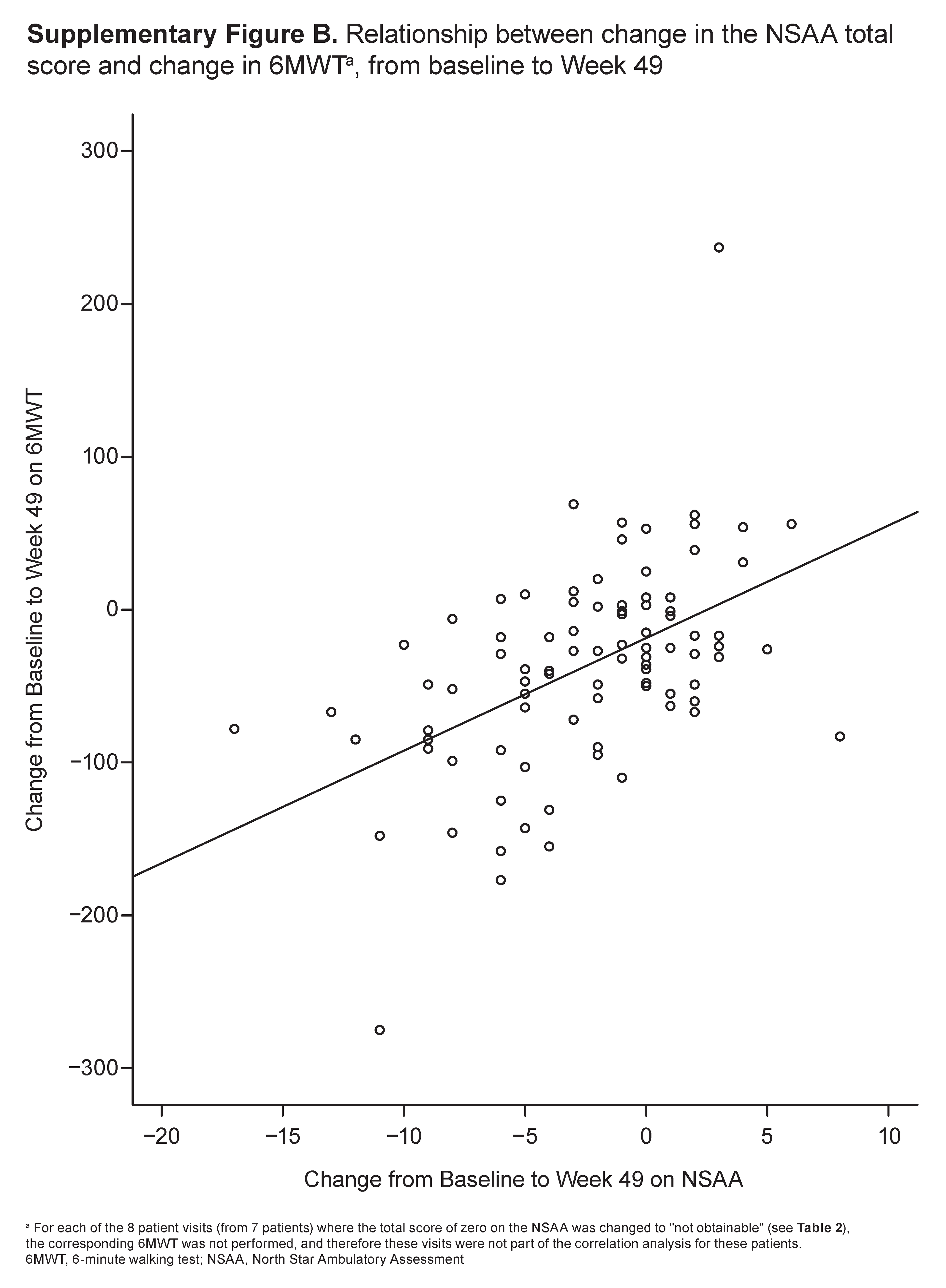

Supplement: S2 Fig — (TIF) [file pone.0272858.s002.tif]
